# The Earliest Evidence of Holometabolan Insect Pupation in Conifer Wood

**DOI:** 10.1371/journal.pone.0031668

**Published:** 2012-02-15

**Authors:** Leif Tapanila, Eric M. Roberts

**Affiliations:** 1 Department of Geosciences, Idaho State University, Pocatello, Idaho, United States of America; 2 Division of Earth Science, Idaho Museum of Natural History, Pocatello, Idaho, United States of America; 3 James Cook University, School of Earth and Environmental Sciences, Townsville, Queensland, Australia; Raymond M. Alf Museum of Paleontology, United States of America

## Abstract

**Background:**

The pre-Jurassic record of terrestrial wood borings is poorly resolved, despite body fossil evidence of insect diversification among xylophilic clades starting in the late Paleozoic. Detailed analysis of borings in petrified wood provides direct evidence of wood utilization by invertebrate animals, which typically comprises feeding behaviors.

**Methodology/Principal Findings:**

We describe a U-shaped boring in petrified wood from the Late Triassic Chinle Formation of southern Utah that demonstrates a strong linkage between insect ontogeny and conifer wood resources. *Xylokrypta durossi* new ichnogenus and ichnospecies is a large excavation in wood that is backfilled with partially digested xylem, creating a secluded chamber. The tracemaker exited the chamber by way of a small vertical shaft. This sequence of behaviors is most consistent with the entrance of a larva followed by pupal quiescence and adult emergence — hallmarks of holometabolous insect ontogeny. Among the known body fossil record of Triassic insects, cupedid beetles (Coleoptera: Archostemata) are deemed the most plausible tracemakers of *Xylokrypta*, based on their body size and modern xylobiotic lifestyle.

**Conclusions/Significance:**

This oldest record of pupation in fossil wood provides an alternative interpretation to borings once regarded as evidence for Triassic bees. Instead *Xylokrypta* suggests that early archostematan beetles were leaders in exploiting wood substrates well before modern clades of xylophages arose in the late Mesozoic.

## Introduction

The evolutionary and ecological origin of the wood boring niche remains cryptic in the field of paleontology, despite extensive body fossil evidence of xylophagous arthropod clades since the middle Paleozoic. Literature on continental borings in wood is sparse and typically non-systematic in comparison to the extensive literature on the paleobiology and ichnology of wood borers in marine settings. Yet, continental wood borings hold great potential to record *in situ* evidence for innovative behaviors that link the metabolic activities of arthropods to plants, which is one of the most significant developments in the Phanerozoic evolution of terrestrial ecosystems.

Wood borings first appear in the Middle Devonian and consist of small anastomosing tunnels similar to those made by modern oribatid mites, which serve as decomposers of cellulose in forest ecosystems (reviewed by [1]). These minute pellet-filled borings dominate the Paleozoic wood boring record, and it is not until the Permian when the first macroscopic borings appear [2]. The oldest continental wood boring trace fossils codified in nomenclature are from the Upper Triassic deposits of western US and Germany [3], [4]. Coincident with the expansion and preservation of conifer forests in the Colorado Plateau, at least five different styles of large borings record the feeding behaviors of insects in woody materials [3]. By the Cretaceous, the wood boring record is more widely recognized globally in terrestrial deposits [5].

This paper describes a large chambered boring discovered in Late Triassic petrified conifers of southern Utah, which we argue is evidence for the larval-pupal-imago activity of a holometabolous insect. The Late Triassic age of this fossil makes it the earliest evidence for pupation in a wood substrate, marking a significant coupling of insect ontogeny with its host conifer. In addition, our interpretation of fossil material from Wolverine Petrified Forest in southern Utah provides a clearer explanation for similar Triassic borings from Arizona's Petrified Forest National Park (PEFO), once regarded as the activity of eusocial bees [6].

### Geological Setting

The Upper Triassic Chinle Formation is one of the best studied and most widely-exposed continental sedimentary successions in the world [7], with high taxonomic diversity of terrestrial vertebrates and abundant fossil wood from the well-known Petrified Forest and Sonsela members [8]. Type and referred specimens include new material collected by the authors from Wolverine Petrified Forest (WPF) of southern Utah.

**Figure 1:**
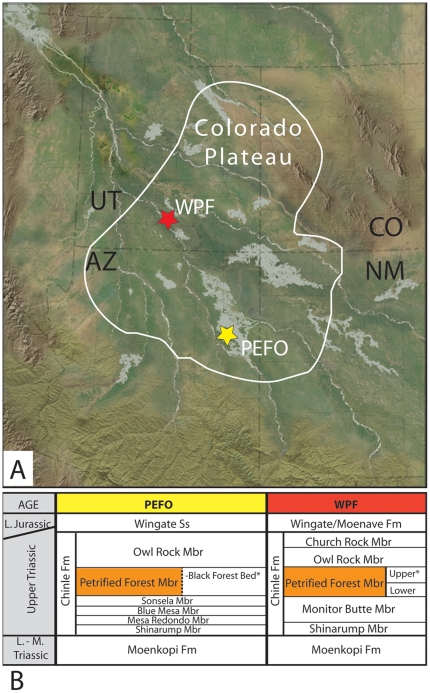
Paleogeographic map and stratigraphy of Colorado Plateau region during Late Triassic. **A.** Map showing Wolverine Petrified Forest (WPF), Utah and Petrified Forest National Park (PEFO), Arizona. Modified after Blakey [44]. **B.** Stratigraphy after [9], [10]; asterisks denote the stratigraphic position of *Xylokrypta* specimens.

WPF is located in the eastern part of Grand Staircase–Escalante National Monument, Utah (Figure 1). Three log samples that preserve wood borings were collected from the Petrified Forest Member in Horse Canyon. The samples are part of an extensive region of well-preserved fossil wood, including 20 m long logs, found in distinctive pinkish colored, 3–4 m thick fluvial sandstone from the top of the Petrified Forest Member. Ash [9] estimated a thickness of ∼550 m for the Chinle Formation in the vicinity of WPF and proposed that the principal wood-bearing pinkish sandstone here is correlative to the Black Forest Bed of the Petrified Forest Member of the Chinle Formation in the northern part of PEFO. If this correlation is correct, the WPF and PEFO borings [10] compared in this study are from equivalent stratigraphic units. Current U-Pb geochronology places the Black Forest Bed in Arizona at 209.93±0.07 Ma [11].

### Institutional Abbreviations

**IMNH-PB**, Idaho Museum of Natural History Paleobotany Collections, Pocatello, Idaho, USA; **UMNH PB**, Natural History Museum of Utah Paleobotany Collections, Salt Lake City, Utah, USA.

## Results

### Systematic Ichnology

***Xylokrypta*** **igen. nov.** urn:lsid:zoobank.org:act:7FC6FF3F-EBE5-4E67-9FFD-88D3371BDE61

Etymology. The ichnogenus is named for *xylo* (Greek) = wood, and *krypta* (Latin) = hidden; feminine.

Diagnosis. Curved boring in wood substrate consisting of two shafts that connect at depth to a central chamber. Meniscate frass fills one shaft and part of the upper central chamber.

Type ichnospecies. *Xylokrypta durossi* isp. nov.

***Xylokrypta durossi*** **isp. nov.** urn:lsid:zoobank.org:act:D15382EE-5654-4C63-B118-093179780A73

**Figure 2:**
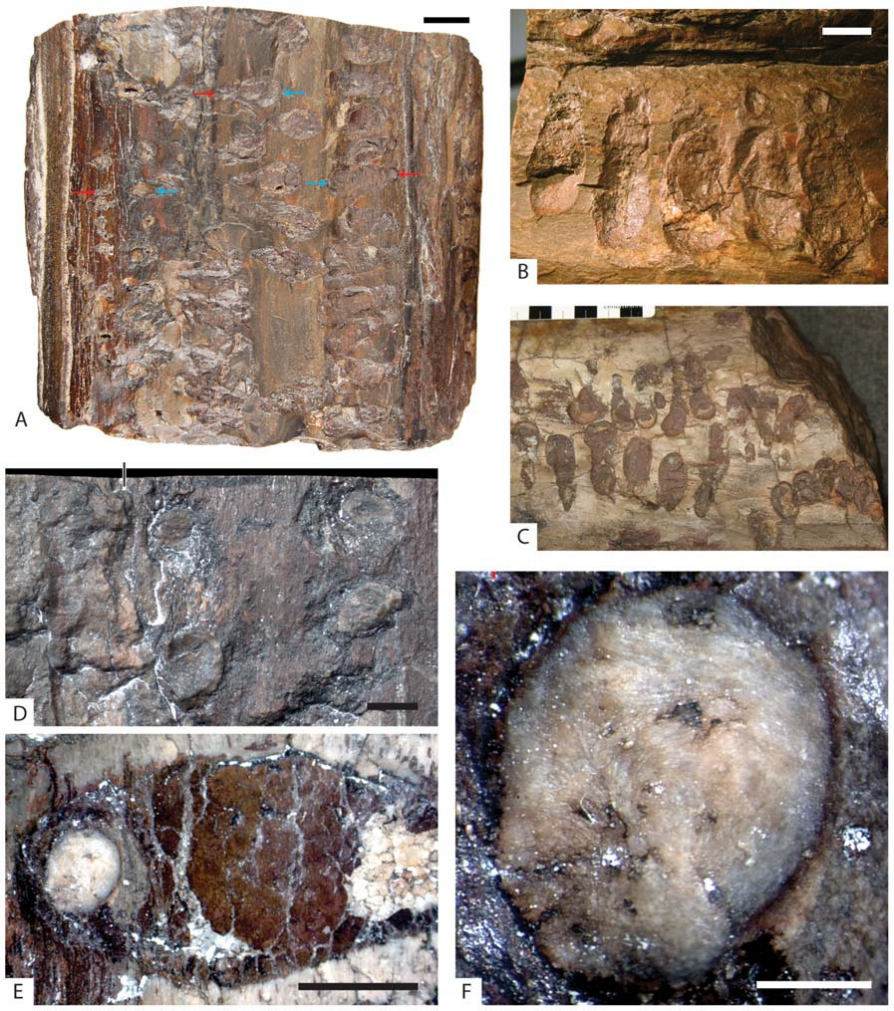
External surface views of *Xylokrypta* in Late Triassic petrified conifer wood. All images of holotype-bearing log UMNH PB 1915, except **C**, IMNH-PB-055/1011. **A.** Xylem cylinder with several series of *Xylokrypta* preserved at various depths of erosion; red arrow marks proximal aperture, blue arrow marks distal opening. Scale bar = 2 cm. **B.** Detail of **A**, with inner bark preserved adjacent proximal apertures at top of image. Scale bar = 1 cm. **C.** Multiple series of deeply eroded *Xylokrypta*. Scale bar intervals = 1 cm. **D.** Two relatively uneroded *Xylokrypta* with proximal (right) and distal (left) apertures and central portion of chamber concealed below surface of wood. Vertical line marks edge of inner bark preserved at left side of image. Scale bar = 5 mm. **E.** Distal part of eroded *Xylokrypta* preserving light-colored frass fill of the distal shaft surrounded by dark-colored frass and hematite of the proximal and chamber cavity. Scale bar = 5 mm. **F.** Detail of **E** showing spiraled organization of coarse tracheid fibers comprising the distal shaft fill. Scale bar = 1 mm.

**Figure 3:**
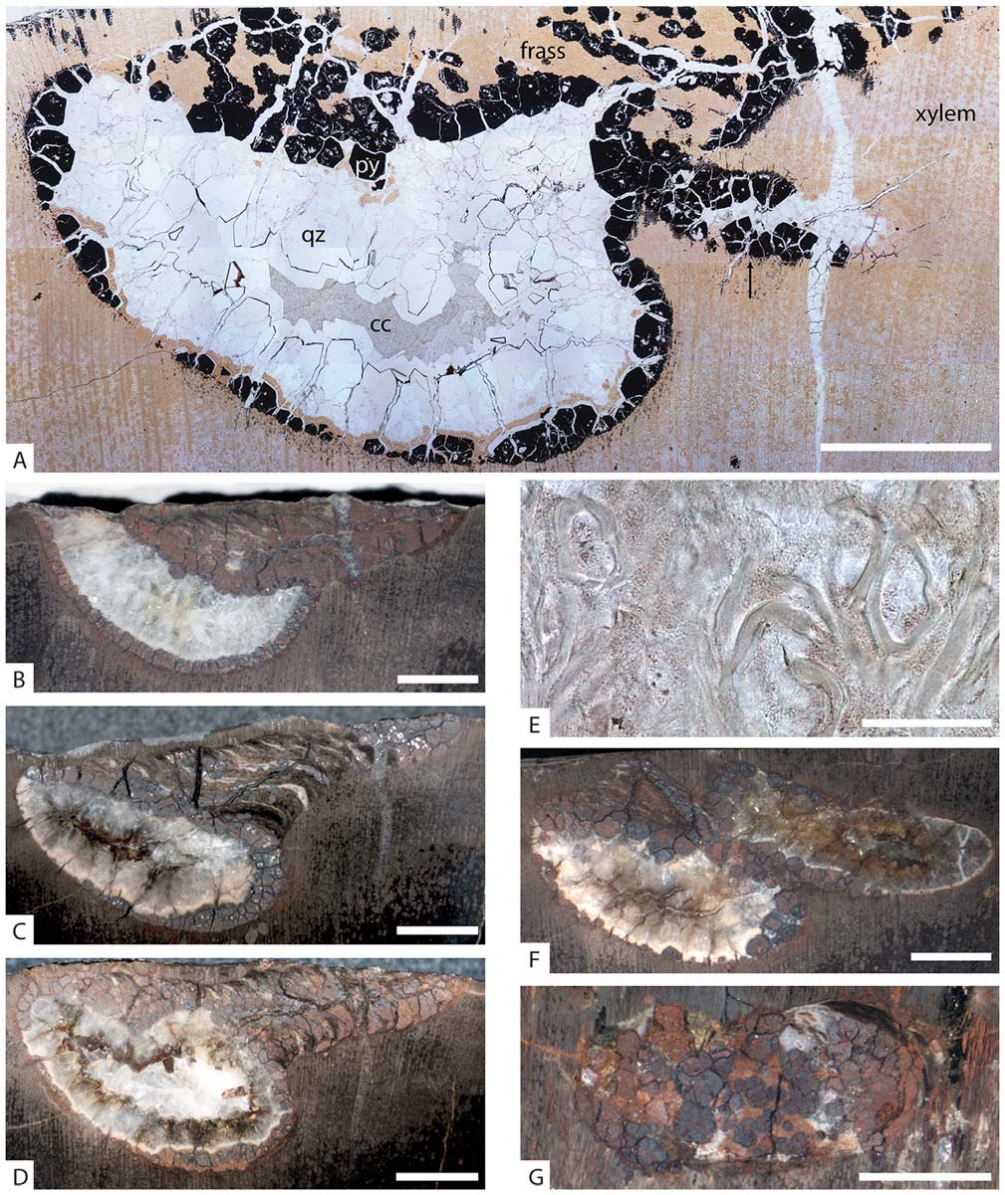
Sections of *Xylokrypta* from UMNH PB 1915. Transverse (**A–F**) and tangential (**G**) sections of the wood substrate. All images oriented with proximal side of boring on the right of image. **A.** Holotype, composite photomicrograph just off-center from midline of boring. Frass and hematite-after-pyrite (**py**) line and fill the upper chamber and proximal shaft; quartz (**qz**) and calcite (**cc**) spar fill the center of the chamber. Margin of adjacent *Xylokrypta* (as in **F**) marked by arrow. **B.** Slab section through midline of boring, bisecting proximal and distal apertures. **C, D.** Holotype, slab sections of boring in making thin section in **A**. Image **C** is photo-reversed to match counterpart in **D**. **E.** Holotype, detail photomicrograph of **A** showing fragmented tracheids found under the word “frass”. **F.** Slab section showing outer portion of chambers of adjacent *Xylokrypta* borings. Holotype is the boring on the left. **G.** Slab section just below wood surface. All scale bars = 5 mm, except **E**, which is 50 microns.

**Figure 4:**
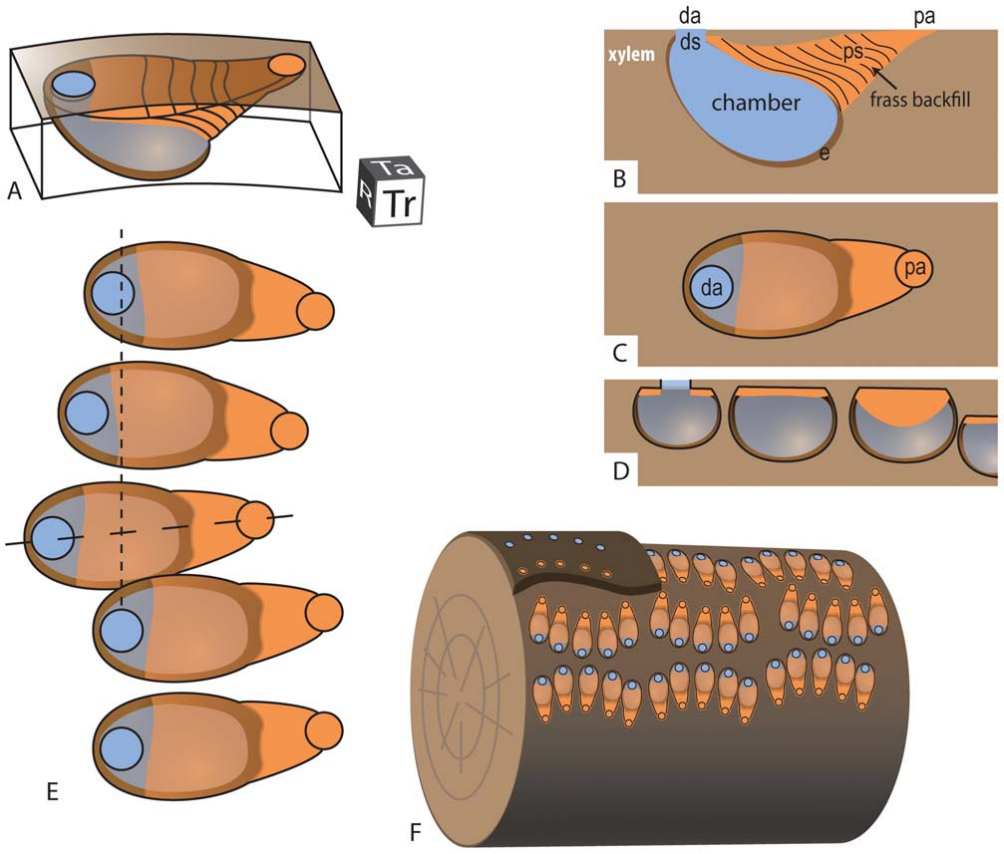
Model of *Xylokrypta* boring. Oblique (**A**), transverse (**B**), tangential (**C**), and radial (**D**) views relative to wood substrate. **E.** Tangential view of typical *Xylokrypta* series. Long dash corresponds to transverse view in **B**, and short dashed line corresponds to radial view in **D**. **F.** Orientation of *Xylokrypta* series on wood cylinder. Decorticated wood reveals central chamber of *Xylokrypta*, which remains concealed if outermost xylem or inner bark are preserved (top left). **Abbreviations: pa**, proximal aperture; **ps**, proximal shaft; **da**, distal aperture; **ds**, distal shaft; **e**, encrustation of hematite-after-pyrite. Planes on cube in **A** marked **R**, radial; **Ta**, tangential; **Tr**, transverse.

Figures 2, 3, and 4.

**Figure 5:**
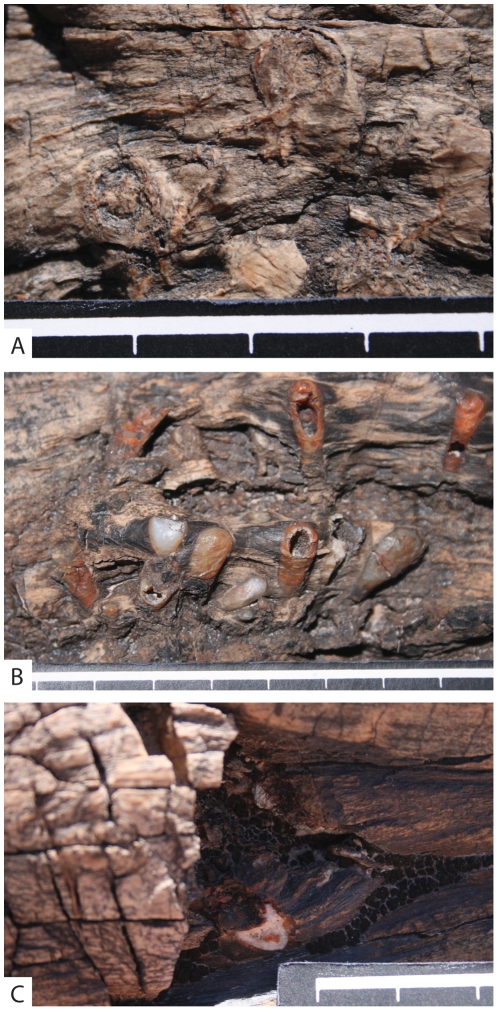
Field photographs of *Xylokrypta* in PEFO specimen. **A.** Apertures containing fill. **B.** Series of *Xylokrypta* with brown lining and sparitized or hollow chambers. **C.** Cross section of boring showing lunate spar at center of boring. All scale bar intervals = 1 cm.

Synonymy. Colonial bees' nests Hasiotis 1997 [6], Figs on p. 22, 23; Bee nest Hasiotis et al. 1998 [12], Fig. 5; cf. *Celliforma* Hasiotis 2003 [13], Fig. 14A–C; Beetle borings Lucas et al. 2010 [10], Figs. 3, 4.

Etymology. The ichnospecies is named in honor of the discoverer of the holotype specimen, Christopher DuRoss.

Type Material. The holotype trace fossil occurs in petrified conifer log, UMNH PB 1915. This conifer specimen (Fig. 2A) preserves a complete secondary xylem cylinder with a small patch of inner bark and no evidence of fungal rot. The cylinder has a diagenetically compressed diameter of 22 cm×18 cm, and is ∼20 cm tall. Borings are exclusively on one half of the circumference of the log. Four sections through the holotype boring are presented (Fig. 3A, C–F), and only the polished surface in Figure 3D was destroyed in making the thin section.

Type Locality, Horizon and Age. The specimen containing the holotype boring is from locality UMNH PB LOC 56, Upper Triassic (Norian) Petrified Forest Member of the Chinle Formation, WPF, Horse Canyon, Utah.

Referred Specimens. At least 40 borings, in addition to the holotype, occur in the petrified conifer log UMNH PB 1915.

Two petrified conifer logs from the same general area and stratigraphy as the type specimen contain borings of the new ichnospecies. IMNH-PB-055/1011 (Fig. 2C) is a cylinder of outermost xylem and has no evidence of fungal rot. The cylinder has a 24 cm diameter and is ∼24 cm tall. Borings are concentrated on one side of the log. IMNH-PB-056/1012 is a deeply eroded specimen of heartwood xylem (10 cm) that shows significant degradation of the wood, likely due to fungal infestation. This piece of wood is too incomplete to determine the extent of boring coverage within the original log.

**Figure 6:**
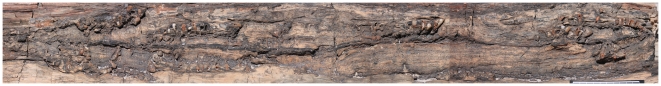
Composite field photograph of PEFO log specimen containing borings diagnosed here as *Xylokrypta*. Borings concentrated along longitudinal furrow. Scale bar at lower right is 10 cm, with 1 cm increments.

Borings in an un-cataloged field specimen at PEFO (Figs. 5, 6) in the Black Forest region north of Interstate 40 are referred to elsewhere [6], [10], [12], [13].

Diagnosis. Central chamber is kidney-shaped. Individual boring oriented with apertures aligned to the transverse plane of the wood cylinder. Series of borings oriented along the longitudinal axis of the wood cylinder, typically numbering five or six.

Description. Three conifer log specimens (?*Araucarioxylon arizonicum*) from WPF contain a total of at least 87 individual boreholes attributed to the new ichnogenus, *Xylokrypta* (Table 1). The conifer logs are quartz permineralized and variably decorticated to reveal natural cross sections through the borings.

**Table 1:** Morphometry of *Xylokrypta* borings from holotype specimen, UMNH PB 1915.

	Proximal width	Distal width	Proximal to distal length
Mean	5.4	10.6	29.2
S.D.	1.4	2.6	4.8
Minimum	3.15	4.75	21.47
Maximum	8.18	14.39	40.01
N	35	40	39

All measurements in mm.

*Xylokrypta durossi* is a curved, roughly U-shaped, boring having two widely-spaced, circular cylindrical shafts that connect below the wood surface to a kidney-shaped chamber. All margins of the boring are sharp, cross-cutting the xylem tissues with minor deformation. The boring penetrates 10–14 mm deep, enough to maintain a 1–2 mm ‘roof’ of xylem above the chamber.

The two shafts of the boring differ in terms of their length, angle of penetration, and infilling material. We use the ichnogenetic terms, *proximal* and *distal*, to describe the two boring shafts and identify directionality in the boring's construction, i.e., from proximal to distal (see Discussion).

The proximal shaft has a minimum diameter of 3.2 mm and connects to the chamber at an acute angle (Fig. 3). The length of the shaft is typically 10 mm, but varies considerably with the angle of excavation and may be as short as 4 mm if the angle is steep (70°), or as long as 15 mm if the angle is shallow (20°). The proximal shaft comprises up to a third of the total length of the boring as it widens toward the chamber. Fragmented tracheids and mineral overgrowths of hematite and quartz occupy the entire region of the proximal shaft. In side view, the tracheid fragments are bundled as meniscae that conform to the curvature of the chamber below, i.e., convex toward the aperture. The fragmented bundles of tracheids in the boring are interpreted as frass, the partially digested excreta of the trace maker.

The kidney-shaped chamber generally measures 18 mm×8 mm×8 mm, with little variation among specimens. Its deepest part is nearest the proximal shaft and inclines upward toward the distal shaft. The interior margin of the chamber is encrusted with euhedral hematite-after-pyrite and quartz cements at the base, and a continuation of fragmentary tracheid fill emanating from the proximal shaft. These materials mantle an inner region of coarse quartz and calcite spar, to form a tubular cast within the chamber that extends to the distal aperture. In some specimens the cast is lunate in cross-section, but more often it is circular.

The distal shaft has a minimum diameter of 4.75 mm and is oriented nearly perpendicular to the much wider chamber below. The maximum length of distal shaft identified so far is only 1 to 2 mm; more often the distal shaft either is not preserved due to erosion or cannot be distinguished from the distal termination of the chamber. Where it has been identified, the distal shaft contains coarse spiral bundles of tracheid fragments, different in appearance to the meniscate bundles found in the proximal shaft and chamber (Fig. 2E, F). In some instances, the distal aperture contains quartz spar that connects below to the tubular spar cast within the chamber.

In plan view, the paired apertures are above the opposing ends of the chamber, and oriented parallel to the transverse plane of the wood cylinder (Fig. 2D). Excavations therefore penetrate the wood in a dominantly radial direction for the shafts and tangential direction for the chamber, i.e., perpendicular to the grain of the wood. Individual *Xylokrypta* borings often are clustered in an arcuate series of five or six, and arranged along the longitudinal axis of the wood cylinder (Fig. 2B, C). Spacing between individual proximal apertures is 4 to 6 mm. Borings do not cross-cut each other. Adjacent borings are accommodated in the wood by having variable length and angle of the proximal shaft or depth of the chamber, sometimes resulting in an en echelon pattern (Fig. 3F).

The holotype log preserves inner bark on either side of the proximal and distal apertures in the xylem and shows no damage from boring (Fig. 2B, D). In IMNH-PB-055/1011, and parts of the holotype-bearing log, the outermost xylem is eroded most deeply in the region containing several distal portions of *Xylokrypta*. In this eroded view, the fossil cavity has the appearance of a flask-shaped boring with a single aperture, but in all other aspects, the boring matches the description of complete *Xylokrypta*, including the presence of meniscate tracheid fill (Fig. 2C).

Remarks. The paired apertures and frass fill of well-preserved *Xylokrypta* distinguish it from all other borings described in wood substrates. Because wood decortication is likely to erode the distal shaft and aperture of the boring, it is necessary to present distinguishing characters of eroded *Xylokrypta* from other single-aperture trace fossils. *Teredolites* Leymerie 1842 [14] differs by having elongate, club-shaped borings that tend to be vermiform throughout their length, do not contain frass, and usually contain a calcitic lining. The narrow, slot-shaped *Asthenopodichnium* Thenius 1979 [15] is uniform in width and free of frass, unlike *Xylokrypta*. Several borings defined as branched tunnels in wood (*Paleoscolytus* Walker 1938 [3]; *Paleoipidus* Walker 1938 [3]; *Paleobuprestis* Walker 1938 [3]; *Cycalichnus* Genise 1995 [5], *Xylonichnus* Genise 1995 [5]; *Stipitichnus* Genise 1995 [5]) can be readily distinguished from the unbranching *Xylokrypta*. Traces ascribed to the ichnofamily Celliformidae Genise 2000 [16] (e.g., *Celliforma* Brown 1934 [17], *Palmiraichnus* Roselli 1987 [18], *Uruguay* Roselli 1938 [19], *Corimbatichnus* Genise and Verde 2000 [20], *Rosellichnus* Genise and Bown 1996 [21], *Ellipsoideichnus* Roselli 1987 [18], *Cellicalichnus* Genise 2000 [16]; and *Brownichnus* Genise 2000 [16]) are yet to be found in wood substrates (*contra* [13]). They can be distinguished morphologically by their lack of curvature at the base of the trace, which is observed in *Xylokrypta*.

### *Xylokrypta* from Petrified Forest National Park

Club-shaped borings described from a log in the Black Forest of Arizona's Petrified Forest National Park have been the subject of dispute since their original designation as the oldest trace fossil evidence for bees [6], [12], [13]. Many researchers have contested the hypothesis of Triassic bees based on body fossil and phylogenetic evidence [2], [16], [22]–[24], but in 2010 Lucas and colleagues [10] reappraised the PEFO borings, disputing many of the primary observations reported by Hasiotis [6]. Limitations of the *in situ* ∼15 m long petrified log prevented a better morphological and process-based alternative explanation for the borings. From our own observations of the PEFO specimen, and in the context of the WPF borings described here, we suggest that the controversial borings from PEFO are *Xylokrypta*.

The PEFO woody stem is decorticated and has the greatest concentration of borings along a 1 m long and 15 cm wide longitudinal furrow in the wood (Figs. 5, 6). Branch knots and at least one fusiform *Polyporites* fungal mass are also sites for borings. In most instances the borings appear to have a single aperture and are flask-shaped, forming clusters of roughly six, and are oriented parallel to the longitudinal axis of the wood cylinder. Borings are filled with orange-brown fine material and white quartz sparite, and are occasionally hollow toward the base of the flask. It is challenging to identify the timing of the borings, whether they were excavated in live or dead wood. Borings appear to be confined to one side of the log, but given the immovable size of the log, thorough inspection of the underside is impossible.

These PEFO borings resemble the deeply eroded *Xylokrypta* of IMNH-PB-055/1011. With the distal portion of the boring eroded, the remaining part of the PEFO borings look flask-shaped. The PEFO borings illustrated by Lucas et al. (Figures 3, 4 in [10]; and additionally photographed here, Figs. 5, 6) document the similarity in morphology to the WPF *Xylokrypta*. With the exception of a few smaller borings, the mean width and length of the PEFO borings overlap WPF specimens. In addition, the frass and sparite fill pattern that we observed in WPF samples is consistent with field observations of the orange-brown fine material and white spar that we observe at PEFO.

Our findings confirm the dissimilarity of the PEFO wood borings with the cell fabrications ascribed to bees (e.g., celliformid burrows) and preclude their use as evidence for Triassic Hymenoptera. It also extends the geographic range for *Xylokrypta* borings and suggests that the morphology recorded by the trace is the result of repeated, patterned behaviors.

## Discussion

### Boring Construction and Ethology

The *Xylokrypta* boring is composed of three distinct modules, starting with the excavation of the proximal shaft, enlargement of a central chamber, and finally, outward evacuation via the distal shaft (Fig. 4). The concentration and meniscate fabric of frass in the proximal shaft and upper chamber support a proximal-to-distal mode of excavation for this boring. In this scenario, wood tissue consumed in excavating the proximal shaft and chamber was excreted and actively backfilled to close-off the proximal aperture. Concealed beneath the thin roof of xylem and plug of frass, the body of the tracemaker occupied the kidney-shaped chamber, now approximated by the tubular cast of sparite. The tracemaker ultimately exited the chamber in the most direct, perpendicular route, sometimes leaving behind a trail of frass (Fig. 2F).

The *Xylokrypta* boring appears to record two chief behavioral modes (ethologies): the shafts demonstrate “feeding+locomotion” by way of frass production, whereas the chamber offered a closed “resting” place protected by backfilled frass. Preservation of bark adjacent to *Xylokrypta* in the holotype shows that *Xylokrypta* does not connect directly to other borings either in the bark or xylem cylinder. Of the two chief ethologies, feeding seems secondary to the construction of the resting chamber. Sap- and heartwood tissues of the xylem cylinder are poor in nutrients in comparison to the phloem and cambium layers [25], and require that xylophages consume large volumes in order to grow. The proximal aperture of *Xylokrypta* is ∼4 mm; an animal with this diameter would minimally benefit from the small volume of wood consumed to make *Xylokrypta* (∼1.0 cm^3^), but would more likely benefit from the generation of a frass-backfilled chamber secluded in a wood log. In addition, the least variable aspect of *Xylokrypta* is the chamber morphology and size. By contrast the proximal shaft ranges widely in length and angle of penetration, possibly in response to limits of accommodation space imposed by adjacent borings.

As a macroscopic continental wood boring, *Xylokrypta* is most consistent with the behaviors of arthropods seeking shelter during a quiescent phase of life. Given the high ratio of metabolic cost to nutritive gain in producing *Xylokrypta*, we suggest that excavation of this boring is most consistent with the preparation of a pupation chamber, and that the modules in its construction record the transition from larva (proximal shaft), pupa (chamber) to imago (distal shaft).

In this scenario, we propose that multiple holometabolan larvae, in their final instar, congregated on the surface of conifer logs. *Xylokrypta* have only been found in serial groupings that are concentrated on one side of a log, suggesting that fallen trees were targeted. Perhaps oriented by the furrow pattern on the bark, small clusters of larvae initiated proximal shafts close to each other. With the deep chamber excavated, the larva backfilled the proximal aperture with frass and began the pupation process. Adult emergence required a short excavation through the remaining wood at the distal end of the chamber. Frass expelled during emergence is preserved in a more pristine spiraled organization than the matted frass packed into the proximal shaft. The tough organic pupal exuvia is not preserved inside the boring, but neither is the cellulose of the permineralized wood. It is remarkable that only frass and sparite fill the chambers, instead of detrital grains of sand from the burial of the log (Figs. 3, 5). Perhaps the exuvia, now replaced with quartz and hematite-after-pyrite, retained the frass from collapsing into the open chamber cavity.

### Potential Triassic Tracemakers

Linking trace fossil with tracemaker is speculative by nature, but worth exploring briefly. Given that *Xylokrypta* is a macroscopic, resting structure excavated in wood, our criteria for identifying plausible tracemakers includes clades within Insecta, Holometabola that (1) have a well-documented body fossil record from the Triassic; and (2) have extant members that are xylophilic. In our attempt to provide the most conservative linkage between trace fossil and tracemaker, we recognize the possibility that these criteria may overlook extinct clades and those with poor fossil records. Given the limited study of Mesozoic continental wood borings and the potential for convergent behaviors amongst ancient clades, we avoid using our trace fossil interpretations as an argument to extend the stratigraphic range of biotaxa.

Most of today's significant wood boring insect clades do not have body fossil records extending as far back as the Triassic period. Among the Coleoptera, the Buprestidae (flat-headed beetles) first occur during the Middle Jurassic [26], the Cerambycidae (round-headed beetles) first occur during the Late Cretaceous [27], [28], and the Curculionidae (weevils, platypodine, bark, and ambrosia beetles) first occur no earlier than the Late Cretaceous based on body fossil and molecular clock estimates [2], [29]. The major holometabolan clades, Hymenoptera (wasps, bees and ants) and Lepidoptera (butterflies and moths) both originated no earlier than the Jurassic Period [2].

The basal coleopteran clade, Archostemata, includes relatively rare beetles in modern forest ecosystems, but they were particularly common during the late Paleozoic and early Mesozoic [2], [30], [31]. Among these, beetles belonging to the Cupedidae first appear in the Middle Triassic and become very common by Late Triassic time [31]. Modern cupedids are xylomycetophagous, deriving nutrients from fungus in rotting wood rather than digesting the wood itself [32], [33]. Crowson [34] and Grimaldi [22] stated that early cupedids were responsible for the Late Triassic borings described by Walker [3] and Hasiotis [6], although neither reported directly on the trace fossil specimens to test their assertions. The association of some *Xylokrypta* borings with fungal degradation of the wood is consistent with the xylomycetophagous habit of cupedids, but not all bored wood specimens show evidence for fungal infestation. Further, the borings in this study are not primarily feeding traces, so the absence of fungal rot is not unexpected.

The literature on modern cupedid behavior is limited, and only one illustration of a cupedid boring, made by Australian *Cupes varians* Lea, could be found for this study [35]. The pupal chamber for *C. varians* consists of a 7 mm×22 mm oval-shaped enlargement of the larval mining tunnel and is isolated by a plug of tightly packed frass. Constructional elements of this pupation chamber bear general resemblance to *Xylokrypta*, except that the latter does not appear to have a direct connection to the zone of larval feeding.

With only one modern species to compare morphology, we instead compared the width of described Triassic cupedid adults to the diameter of the distal aperture in *Xylokrypta*, which we interpret is the emergent cavity for the adult tracemaker. If Cupedidae produced *Xylokrypta*, we expect that Triassic members of this clade should have body dimensions that are consistent with the boring. The minimum distal width of well-preserved *Xylokrypta* is 4.75 mm (Fig. 2D), and the narrowest plug of distal frass has a diameter of 3.5 mm (Fig. 2F). We regard these distal dimensions of *Xylokrypta* as the best approximation for the maximum width of the tracemaker. In addition, the adult animal should be able to fit inside the *Xylokrypta* chamber, which has a typical length of 18 mm.

**Figure 7:**
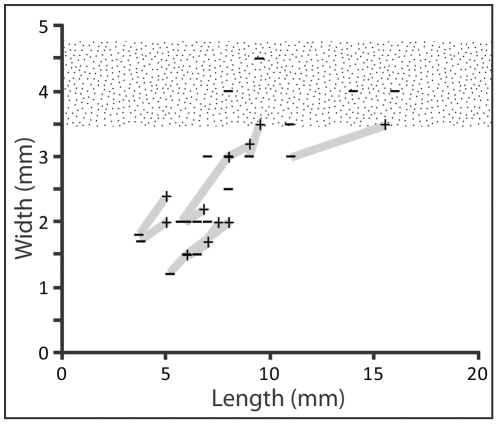
Length and width measurements of 21 species of cupedid beetle (Archostemata: Cupedidae). Minimum (−) and maximum (+) dimensions from individual taxa connected by grey line. Stippled field denotes diameter range of distal aperture in *Xylokrypta*.

North American insect collections poorly record the Norian time interval, precluding coeval comparison with the Chinle Formation. Also, cupedid larvae are not described from the Triassic fossil record. Our analysis of Triassic Cupedidae includes 22 species from upper Ladinian to lower Carnian (∼228 Ma) deposits in Asia, South Africa and Australia (Table 2, Fig. 7) [36], [37], [38]. Nearly all Carnian cupedid adults range in width from 1.2–4.5 mm and body lengths of 3.7–16 mm; one large species, *Moltenocupes townrowi* Zeuner 1961 [36], is a significant outlier with a length of 23 mm and estimated width of 7.5 mm.

**Table 2:** Morphometry of Triassic cupedid beetles (Archostemata: Cupedidae).

	Body length		Body width		Reference
	Min	Max	Min	Max	
***Platycupes dolichocerus***	**8**		**4.0**		[38]
***Platycupes major***	**16**		**4.0**		[38]
*Platycupes pusillus*	3.7	5	1.8	2.4	[38]
*Platycupes reticulatus*	6	6.8	2.0	2.2	[38]
***Platycupes sogdianus***	**9**	**9.5**	**3.0**	**3.5**	[38]
*Triadocupes ellipticus*	3.8	5	1.7	2	[38]
*Triadocupes ferghanensis*	5.7	8	2.0	3	[38]
*Triadocupes latus*	7	7.3	3.0		[38]
*Pterocupes antennatus*	6	7	1.5	1.7	[38]
*Pterocupes leptocerus*	7		2.0		[38]
***Cupesia monilicornia***	**9.5**		**4.5**		[38]
***Procupes mandibularis***	**14**		**4.0**		[38]
*Asimma rara*	6.5		2.0		[38]
*Kirghizocupes cellulosus*	6.5	7.5	1.5	2	[38]
*Moltenocupes townrowi*	23		7.5*		[36]
***Lithocupes incertus***	11*		**3.5**		[38]
*Notocupoides capitatus*	8	9	3.0	3.2	[38]
*Notocupoides fasciatus*	5.2	6	1.2	1.5	[38]
*Notocupoides triassicus*	8		2.5		[38]
*Rhabdocupes longus*	11	15.5	3.0	3.5	[38]
*Rhabdocupes minor*	6	8	1.5	2	[38]
***Mesothoris clathrata***	11*		**3.6**		[37]

Taxa in bold have dimensions that overlap with the distal aperture and chamber size of *Xylokrypta*. All measurements in mm. Asterisk denotes that the dimension was extrapolated from length∶width ratio on Figure 7. Beetle taxa from Carnian [36], [37] and Ladinian–Carnian [38] deposits.

Seven of the 22 Triassic adult cupedids are size-consistent with *Xylokrypta*, having widths between 3.5–4.75 mm (i.e., the range of the distal aperture). These seven beetle taxa have body lengths from 8–16 mm, and could have been accommodated by the ∼18 mm-long *Xylokrypta* chamber. Given that one third of the cupedid species we analyzed are size-consistent with *Xylokrypta*, we conclude that Cupedidae are the most plausible candidate tracemakers based on the known body fossil record.

### Implications for Adaptation

Triassic *Xylokrypta* are the oldest and most complete trace fossils in any substrate to show the distinct pupation behaviors of holometabolous insects. These traces become more common in Cretaceous soil and bone substrates [39], [40], with the great diversification of holometabolans at that time. The Late Triassic origin of pupation in wood corresponds with an increase in wood utilization by arthropods, especially insects (e.g., [3]).

Three adaptive advantages for pupating in wood include (1) proximity to food source, (2) protection from enemies, and (3) control of microenvironment during pupation. Frass preserved inside the pupation cavity demonstrates that the tracemaker of *Xylokrypta* was capable of biting and partially digesting xylem tissues, suggesting that the tracemaker was a xylo- or xylomycetophage. The primary feeding trace of the *Xylokrypta*-producing larva has yet to be identified; perhaps it is one of the borings described by Walker, or the larva may have consumed other parts of the plant near the site of pupation. Protection from predation while undergoing quiescent pupation seems a likely advantage, although there is little documentation of increased predation pressure on insects living in soils during the Late Triassic [41]. Last, the microenvironmental advantage of a wooden pupation chamber would have stabilized temperature and enhanced humidity saturation; both factors are known to be important controls on the duration and timing of pupation as observed in modern wood boring and gall-forming insects (e.g., [42]). With a mean annual temperature of 29°C and precipitation of 400–600 mm [43], the hot, dry climate of the Colorado Plateau region during the deposition of the upper Chinle Formation could have made pupation chambers in wood advantageous.

## Materials and Methods

### Field Methods and Preparation

Holotype and referred specimens from WPF were collected in the field under permits GSENM UT-09-030-01-G, UT06-007S-GS. Permineralized wood was prepared using lapidary saws to cut polished slab and petrographic thin sections. The large un-cataloged PEFO specimen was studied in the field and photo-documented. Morphometry of the boring was performed using digital calipers for external dimensions and digital photomicrographs for slab and thin section preparations.

### Nomenclatural Acts

The electronic version of this document does not represent a published work according to the International Code of Zoological Nomenclature (ICZN), and hence the nomenclatural acts contained in the electronic version are not available under that Code from the electronic edition. Therefore, a separate edition of this document was produced by a method that assures numerous identical and durable copies, and those copies were simultaneously obtainable (from the publication date noted on the first page of this article) for the purpose of providing a public and permanent scientific record, in accordance with Article 8.1 of the Code. The separate print-only edition is available on request from PLoS by sending a request to PLoS ONE, Public Library of Science, 1160 Battery Street, Suite 100, San Francisco, CA 94111, USA along with a check for $10 (to cover printing and postage) payable to “Public Library of Science”.

In addition, this published work and the nomenclatural acts it contains have been registered in “http://zoobank.org”, the proposed online registration system for the ICZN. The ZooBank LSIDs (Life Science Identifiers) can be resolved and the associated information viewed through any standard web browser by appending the LSID to the prefix “http://zoobank.org/”. The LSID for this publication is: urn:lsid:zoobank.org:pub:9FE09CA5-98C0-4D6F-8960-B411A5C27904.
